# Adverse body measurements are superior to sarcopenia-associated measurements in predicting chronic diseases

**DOI:** 10.1038/s41598-021-85316-0

**Published:** 2021-04-08

**Authors:** Pei-Ju Liao, Yu-Ching Lin, Ming-Kuo Ting, I.-W.en Wu, Shuo-Wei Chen, Ning-I. Yang, Kuang-Hung Hsu

**Affiliations:** 1 Master Degree Program in Healthcare Industry, Chang Gung University, Taoyuan, Taiwan; 2 Division of Medical Imaging, Chang Gung Memorial Hospital, Keelung and Chang Gung University, Taoyuan, Taiwan; 3 Division of Endocrinology and Metabolism, Chang Gung Memorial Hospital, Keelung, Taiwan; 4 Division of Nephrology, Chang Gung Memorial Hospital, Keelung, Taiwan; 5 Division of Gastroenterology and Hepatology, Chang Gung Memorial Hospital, Keelung, Taiwan; 6 Division of Cardiology, Chang Gung Memorial Hospital, Keelung, Taiwan; 7 Healthy Aging Research Center, Chang Gung University, Taoyuan, Taiwan; 8 Laboratory for Epidemiology, Department of Health Care Management, Chang Gung University, Kwei-Shan, No. 259, Wen-Hwa 1st Road, Taoyuan, 333 Taiwan; 9 Department of Emergency Medicine, Chang Gung Memorial Hospital, Taoyuan, Taiwan; 10 Department of Urology, Chang Gung Memorial Hospital, Taoyuan, Taiwan; 11 Research Center for Food and Cosmetic Safety, College of Human Ecology, Chang Gung University of Science and Technology, Taoyuan, Taiwan; 12 Department of Safety, Health and Environmental Engineering, Ming Chi University of Technology, New Taipei City, Taiwan

**Keywords:** Predictive markers, Predictive markers, Preventive medicine

## Abstract

Few studies have demonstrated an association of sarcopenia-associated body measurements with chronic diseases through a comprehensive methodology. This study aims to examine the relationship between sarcopenia-associated body measurements and chronic diseases. This is a cohort study. We recruited 316 community dwellers, including 76 patients with sarcopenia and 240 controls, and obtained their body measurements associated with sarcopenia. We collected three-dimensional anthropometric body-surface measurements from 11,158 participants during 2000–2008 and followed up this cohort for 15 years to examine the association of these measurements with the risk of chronic diseases such as hypertension, type 2 diabetes mellitus (T2DM), heart disease, and nephrotic syndrome. Univariate analysis, canonical correlation, and Cox regression analysis were performed to explore the associations. Decreased waist width, upper left arm circumference, and left thigh circumference were significantly associated with sarcopenia. The adverse body measure score (ABMS) was derived by combining significant measurements, namely left upper arm circumference, waist width, and left thigh circumference, and used to predict the risk of hypertension, T2DM, heart diseases, and nephrotic syndrome. A positive association was observed between the ABMS and chronic diseases. Considering the first quartile of the ABMS as a reference, we determined hazard ratios of 2.259, 2.495, 1.332, and 1.595 for hypertension, T2DM, heart disease, and nephrotic syndrome, respectively, in the fourth quartile. Chronic diseases were more strongly associated with the ABMS than with sarcopenia-related body measurements alone. A high ABMS, which includes higher upper arm circumference, higher waist width, and lower thigh circumference, can significantly predict chronic diseases.

## Introduction

Decreased muscle mass is associated with advanced age, an inactive lifestyle, and inadequate nutrition. Sarcopenia is generally characterized by the co-occurrence of muscle reduction and dysfunction^1,2^. Sarcopenia results in adverse health outcomes, including increased mortality and long hospitalization, and is recognized as a systemic condition associated with metabolic syndrome, diabetes, cardiovascular disease, depression, and possibly cognitive impairment^3–9^. The prevalence of sarcopenia increases with age and was approximately 13%, ranging from 4.6 to 67%, in the older community-dwelling population^1,10,11^.

Sarcopenia can be classified into three stages: presarcopenia (low muscle mass), sarcopenia (low muscle mass along with either low muscle strength or poor physical function), and severe sarcopenia (low muscle mass, low muscle strength, and poor physical function)^1^. However, it is not feasible to apply the aforementioned classification criteria for sarcopenia to a large population in an epidemiological study. In addition, from a clinical perspective, the presence of an adverse body shape or a combination of body measures, including sarcopenic obesity, a large waist-to-thigh ratio, and lean lower limbs is the most concerning^12,13^. Specific body measurements can provide useful information for predicting adverse outcomes in the older community-dwelling population. However, limited evidence is available in this context.

Sarcopenia and sarcopenic obesity are associated with chronic diseases. Cardiovascular disease, dementia, and type II diabetes mellitus (T2DM) were reported to frequently co-occur with sarcopenia in older adults and have common risk factors such as aging, an adverse body shape, malnutrition, and an inactive lifestyle^9^. In addition, studies have suggested some potential consequences of sarcopenia including depression, physical dysfunction, dependency, and mortality^1,9,14,15^. These outcomes are correlated with body measurements such as lean lower limbs and visceral fat accumulation.

Some body measurements may be highly correlated with sarcopenia; however, obtaining body measurements is important for early prediction and prevention of this condition. Therefore, body measurements related to sarcopenia are considered crucial biomarkers of poor health outcomes. Three-dimensional techniques can be used to determine body composition at a low cost; such techniques are highly applicable in healthy individuals. Epidemiological studies have used anthropometric measurements, such as body mass index (BMI), waist circumference (WC), and waist-to-hip ratio (WHR), and have also explored different new body measurements for predicting chronic diseases. Studies have debated whether existing or innovative body measurements, such as BMI, WC, thigh circumference, WHR, and waist-to-thigh ratio, correlate better with selected diseases, such as T2DM, hypertension, dementia, and cancer^12,16,17^. Whether sarcopenia-related body measurements are associated with poor health outcomes is still imperfectly understood. Therefore, in the present study, we determined sarcopenia-related body measurements of community dwellers and conducted a 10-year follow-up of the cohort to investigate the causation.

## Methods

### Study participants and data collection

All participants provided written informed consent for the procedure and the use of their data. The study protocol is in accordance with the ethical guidelines of the 1975 Declaration of Helsinki, and this study was approved by the Institutional Review Board of Chang Gung Medical Foundation (approval number: 201600934B0, 201802378B0A3, and 201801704B0). The study population was enrolled from a community located in northern Taiwan. In total, 316 community dwellers, including 76 patients with sarcopenia and 240 individuals without sarcopenia, were recruited in this study. The health consequences of sarcopenia-related body measurements were investigated using a cohort setup from 2000 onward. We recruited a total of 11,158 participants (5,872 men and 5,286 women) from the department of health examination from among those who were seeking an annual health checkup at a teaching hospital in northern Taiwan. A total of 7,797 participants without hypertension, T2DM, heart diseases, or nephrotic syndrome were included; the mean age of study participants was 49.17 (range 18–91) years. The average age of men and women was similar (49.20 and 49.13 years, respectively). Furthermore, the proportion of men (52.3%) and women (47.7%) in the study was similar. The study follow-up lasted for 17 years and 10 months, with an average of 14.55 years, from February 2000 to December 2017. The total follow-up period was 113,483.97 person-years, of which 58,170.90 person-years were for men and 55,313.07 person-years for women.

Information regarding confounders, namely age, sex, smoking, alcohol drinking, betel nut chewing, occupation, education, marriage, diet, and personal history of diseases, such as diabetes, hypertension, heart disease, renal diseases, liver cirrhosis, and chronic hepatitis, was collected using a questionnaire and confirmed through a medical chart review. T2DM, hypertension, heart disease, and nephrotic syndrome were defined based on International Classification of Diseases, Ninth Revision, Clinical Modification (ICD-9-CM) codes 250.xx (excluding 250.x1); 401–405; 390–398, 410–414, and 420–429; and 580–589, respectively. In the study population, 25.5% were smokers. The prevalence of smoking was lower in women (5.2%) than in men (44.0%). Furthermore, 26.4% of participants regularly consumed alcohol. The prevalence of alcohol drinking was lower in women (8.6%) than in men (42.6%).

### Anthropometric parameters

Sarcopenia was assessed on the basis of muscle mass, muscle strength, and activity function. Muscle mass was evaluated using a single dual-energy fan-beam X-ray absorptiometer (DXA, GE medical system, Lunar iDXA, Madison, WI, USA). Scan modes (standard, thin, or thick) were automatically selected by the scanner software depending on body size and BMI. Scans were analyzed using enCORE Software, version 15 (GE Lunar). Muscle strength was measured on the basis of handgrip strength by using a single dynamometer. We measured the handgrip strength of the dominant hand. A walking speed of < 0.8 m/s for a total distance of 4 m was defined as insufficient activity function.

According to the Asian Working Group for Sarcopenia guidelines, sarcopenia was defined as the presence of low muscle mass along with low muscle strength. Furthermore, low muscle mass without low muscle strength was defined as presarcopenia. The recommended cutoff points for low muscle mass, determined using a height-adjusted appendicular skeletal muscle index, are 7.0 kg/m^2^ for men and 5.4 kg/m^2^ for women. A handgrip strength of 26 and 18 kg was considered low for men and women, respectively^18^. Participants with presarcopenia and sarcopenia were pooled together and compared with controls to examine differences between body surface measurements.

Three-dimensional body surface measurements were performed using methods developed by the laboratory of whole-body 3D laser scanning in the study teaching hospital^19^. Approximately 280 standardized landmarks from the body surface were used to construct 35 body measurements for the head and neck, trunk, upper limbs, and lower limbs. In addition to body height and body weight, 35 measurements were included in this study, namely 4 measurements of the head and neck (head volume, head surface area, head circumference, and neck circumference), 11 measurements of the trunk and hip (waist profile area, WC, waist width, breast profile area, breast width, breast circumference, hip profile area, hip circumference, hip width, trunk surface area, and trunk volume), 10 measurements of the upper limbs (left arm volume, left arm surface area, left arm length, left forearm circumference, left upper arm circumference, right arm volume, right arm surface area, right arm length, right forearm circumference, and right upper arm circumference), and 10 measurements of the lower limbs (left leg volume, left leg surface area, left leg circumference, right leg volume, right leg surface area, right leg circumference, left thigh circumference, right thigh circumference, left leg length, and right leg length).

### Statistical analyses

Numerical variables are displayed as the mean ± standard deviation, and categorical variables are presented as the frequency and percentage. We used the two-sample independent *t* test and chi-square test to determine differences between continuous and categorical variables, respectively. Three-dimensional body surface measurements were categorized according to different body regions, including the whole body, head and neck, trunk, hip, upper limbs, and lower limbs. We performed the two-sample *t* test to examine differences in body surface measurements between cases and controls. To prevent collinearity in the regression analysis, one statistically significant body measurement (*p* < 0.05) was selected from each region of the body for subsequent multivariable analyses. We performed a canonical correlation analysis to construct a linear equation of selected body measures based on the highest strength of the association with multiple chronic diseases. The linear equation of selected body measures was used as an adverse body measure score (ABMS) to validate the prediction of chronic diseases by using a prospective cohort. The sarcopenia-associated body measure score (SBMS) was constructed through the backward model selection of logistic regression by using sarcopenia as the dependent variable and selected body measures examined in the univariate analysis as independent variables. We used a Cox proportional hazards regression model to examine the strength of the association between both the body measure scores and the incidence of chronic diseases, namely T2DM, hypertension, cardiovascular disease, and renal syndrome, expressed as the hazard ratio after adjusting for the following variables: age, sex, marital status, educational level, occupation, cigarette smoking, alcohol drinking, and betel nut chewing. Statistical analysis was performed using SAS 9.4.

## Results

The basic sociodemographic, lifestyle, and disease status variables did not significantly differ between cases and controls in the study cohort. Among study participants, approximately 46% were aged > 60 years, 61–70% were women, 42–35% had a senior high school education, 85–88% were employed in commerce or self-employed, 92–95% were married, 56% had low annual income (< US$10,000), 12–14% were smokers, 11–17% were alcohol drinkers, 2–4% were betel nut chewers, 40–53% were tea drinkers, 51–59% were coffee drinkers, 56–65% exercised regularly, 15–25% had hypertension, 9–10% had T2DM, 3–8% had heart diseases, and 6–13% had kidney diseases (Table 1).

**Table 1:** Distribution of basic characteristics between participants with sarcopenia and controls.

Basic sociodemographic	Cases (n = 76)		Controls (n = 240)		Chi-square *p* value	OR	95% CI
	n	(%)	n	(%)			
**Age**							
< 60	41	(53.95)	129	(53.75)	0.976	1.008	(0.601,1.691)
> = 60	35	(46.05)	111	(46.25)		1.000	–
**Gender**							
Male	29	(38.16)	73	(30.42)	0.2639	1.412	(0.824, 2.418)
Female	47	(61.84)	167	(69.58)		1.000	–
**Education attainment**							
Junior high school or below	22	(28.95)	83	(34.58)	0.5146	1.000	–
Senior high school	32	(42.11)	84	(35.00)		1.437	(0.772, 2.677)
College or above	22	(28.95)	70	(29.17)		1.186	(0.606, 2.320)
**Occupation**							
Government	5	(6.58)	21	(8.75)	0.8314	1.000	–
Labor workers	4	(5.26)	13	(5.42)		1.292	(0.292, 5.708)
Commerce, self-employees	67	(88.16)	206	(85.83)		1.366	(0.496, 3.763)
**Marital status**							
Unmarried	5	(6.58)	11	(4.58)	0.5373	1.481	(0.498, 4.406)
Married	70	(92.11)	228	(95.00)		1.000	–
Unknown	1	(1.32)	1	(0.42)		3.257	(0.201, 52.750)
**Income**							
< USD 10,000	43	(56.58)	135	(56.25)	0.7733	1.000	–
USD 10,000 ~ 16,667	10	(13.16)	40	(16.67)		0.785	(0.362, 1.701)
USD 16,667 ~ 25,000	10	(13.16)	19	(7.92)		1.652	(0.714, 3.824)
USD 25,000–33,333	6	(7.89)	24	(10.00)		0.785	(0.301, 2.046)
> USD 33,333	3	(3.95)	7	(2.92)		1.346	(0.333, 5.431)
Unknown	4	(5.26)	13	(5.42)		0.966	(0.299, 3.119)
**Lifestyle variables**							
**Cigarette smoking**							
No	65	(85.53)	210	(87.50)	0.8023	1.000	–
Yes	11	(14.47)	30	(12.50)		1.185	(0.563, 2.495)
**Alcohol drinking**							
No	67	(88.16)	200	(83.33)	0.4061	1.000	–
Yes	9	(11.84)	40	(16.67)		0.672	(0.310, 1.457)
**Betel nut chewing**							
No	73	(96.05)	235	(97.92)	0.6294	1.000	–
Yes	3	(3.95)	5	(2.08)		1.932	(0.451, 8.278)
**Tea drinking**							
No	45	(59.21)	113	(47.08)	0.0871	1.000	–
Yes	31	(40.79)	127	(52.92)		0.613	(0.363, 1.034)
**Coffee drinking**							
No	37	(48.68)	98	(40.83)	0.2834	1.000	–
Yes	39	(51.32)	142	(59.17)		0.727	(0.433, 1.221)
**Exercise**							
No	33	(43.42)	83	(34.58)	0.2089	1.000	–
Yes	43	(56.58)	157	(65.42)		0.689	(0.407, 1.165)
**Diseases**							
**Hypertension**							
No	63	(82.89)	176	(73.33)	0.2214	1.000	–
Yes	12	(15.79)	61	(25.42)		0.55	(0.278, 1.088)
Unknown	1	(1.32)	3	(1.25)		0.931	(0.095, 9.117)
**Type 2 DM**							
No	68	(89.47)	212	(88.33)	0.9544	1.000	–
Yes	7	(9.21)	25	(10.42)		0.873	(0.362, 2.108)
Unknown	1	(1.32)	3	(1.25)		1.039	(0.106, 10.156)
**Heart diseases**							
No	72	(94.74)	217	(90.42)	0.4393	1.000	–
Yes	3	(3.95)	20	(8.33)		0.452	(0.131, 1.566)
Unknown	1	(1.32)	3	(1.25)		1.005	(0.103, 9.810)
**Kidney diseases**							
No	65	(85.53)	221	(92.08)	0.1987	1.000	–
Yes	10	(13.16)	16	(6.67)		2.125	(0.920, 4.908)
Unknown	1	(1.32)	3	(1.25)		1.133	(0.116, 11.080)

When comparing presarcopenia and sarcopenia cases with controls, we found that all significant body measurements were negatively correlated. Three body measures, namely left upper arm circumference (odds ratio [OR] 0.683; 95% confidence interval [CI] 0.605–0.772), waist width (OR 0.713; 95% CI 0.636–0.8), and left thigh circumference (OR 0.745; 95% CI 0.681–0.816), were found to be significant in the multiple logistic regression analysis (Table 2). We performed a canonical correlation analysis to construct a linear equation of significant body measures, namely left upper arm circumference, waist width, and left thigh circumference, in relation to selected chronic diseases, namely T2DM, hypertension, heart diseases, and renal syndrome. The combination of three body measures (0.00365 × left upper arm circumference + 0.33597 × waist width − 0.10713 × left thigh circumference) was constructed as adverse body measures score (ABMS). The three body measures (17.3571 − 0.147 × left upper arm circumference − 0.1595 × waist width − 0.194 × left thigh circumference) were combined to measure the SBMS (Table 3).

**Table 2:** Descriptive statistics of 3D surface body measures between participants with sarcopenia/presarcopenia and controls.

	Cases (n = 76)	Controls (n = 240)	Univariate OR(95%CI)	Multiple OR(95%CI)
**Whole body**				
Height	160.69 ± 8.49	158.77 ± 8.01	1.029 (0.997, 1.062)	1.053 (1.003, 1.106)
Weight	53.66 ± 9.10	63.36 ± 11.62	0.899 (0.868, 0.932)	0.838 (0.797, 0.882)
BMI	20.65 ± 2.42	25.04 ± 3.50	0.545 (0.466,0.637)	0.480 (0.396, 0.582)
**Head and neck**				
Head circumference (cm)	57.31 ± 3.16	58.26 ± 2.59	0.884 (0.804, 0.971)	0.854 (0.771, 0.946)
Head surface area (cm^2^)	1504.70 ± 481.35	1460.06 ± 142.79	1.055 (0.960, 1.160)	1.053 (0.954, 1.161)
Head volume (cm^3^)	4925.16 ± 520.80	5094.30 ± 600.65	0.949 (0.905, 0.995)	0.906 (0.855, 0.959)
Neck circumference (cm)	37.15 ± 4.65	39.18 ± 4.44	0.902 (0.848, 0.959)	0.853 (0.793, 0.918)
**Trunk**				
Chest width (cm)	29.72 ± 2.78	31.65 ± 2.89	0.774 (0.694, 0.864)	0.699 (0.615, 0.795)
Chest circumference (cm)	90.34 ± 8.85	98.15 ± 11.01	0.923 (0.894, 0.952)	0.916 (0.886, 0.947)
Chest sectional area (cm^2^)	6148.49 ± 1226.19	6963.77 ± 1393.64	0.952 (0.931, 0.973)	0.940 (0.916, 0.963)
Waist width (cm)	28.26 ± 2.67	30.89 ± 3.18	0.740 (0.665, 0.823)	0.713 (0.636, 0.800)
Waist circumference (cm)	74.70 ± 7.97	81.02 ± 9.82	0.923 (0.893, 0.954)	0.911 (0.879, 0.945)
Waist sectional area (cm^2^)	6039.81 ± 1283.90	6989.36 ± 1418.24	0.945 (0.924, 0.967)	0.936 (0.913, 0.960)
Trunk surface area (cm^2^)	7028.05 ± 1083.28	11,656.68 ± 55,906.93	0.955 (0.933, 0.978)	0.948 (0.924, 0.973)
Trunk volume (cm^3^)	37,400.9 ± 7197.27	45,047.98 ± 26,972.78	0.994 (0.991, 0.997)	0.991 (0.988, 0.995)
**Hip**				
Hip width (cm)	32.79 ± 2.07	34.66 ± 2.48	0.684 (0.593, 0.789)	0.671 (0.577, 0.781 )
Hip circumference (cm)	85.89 ± 5.81	90.63 ± 7.40	0.889 (0.847, 0.934)	0.891 (0.847, 0.936)
Hip sectional area (cm^2^)	599.87 ± 116.49	718.04 ± 134.36	0.990 (0.986, 0.993)	0.989 (0.986, 0.993)
**Upper limbs**				
**Upper arm circumference (cm)**				
Left	27.97 ± 2.64	30.64 ± 3.25	0.73 (0.657, 0.812)	0.683 (0.605, 0.772)
Right	27.85 ± 2.85	30.51 ± 3.24	0.73 (0.655, 0.813)	0.672 (0.592, 0.764)
**Forearm circumference (cm)**				
Left	18.84 ± 2.36	20.42 ± 2.58	0.762 (0.676, 0.858)	0.716 (0.625, 0.819)
Right	19.33 ± 2.26	20.80 ± 2.60	0.774 (0.686, 0.873)	0.706 (0.612, 0.815)
**Arm length (cm)**				
Left	51.63 ± 3.66	51.46 ± 3.62	1.013 (0.943, 1.088)	1.011 (0.934, 1.094)
Right	51.75 ± 3.63	51.55 ± 3.59	1.015 (0.944, 1.091)	1.014 (0.936, 1.098)
**Arm surface area (cm**^**2**^**)**				
Left	1141.99 ± 126.66	1187.64 ± 128.31	0.997 (0.995, 0.999)	0.996 (0.994, 0.999)
Right	1193.50 ± 129.95	1243.45 ± 132.30	0.997 (0.995, 0.999)	0.996 (0.994, 0.999)
**Arm volume (cm**^**3**^**)**				
**Lower limbs**				
Left	1887.11 ± 261.49	2110.50 ± 360.71	0.998 (0.997, 0.999)	0.997 (0.996, 0.998)
Right	1901.16 ± 266.18	2115.20 ± 360.51	0.998 (0.997, 0.999)	0.997 (0.996, 0.998)
**Thigh circumference (cm)**				
Left	47.27 ± 3.71	51.89 ± 4.71	0.763 (0.704, 0.827)	0.745 (0.681, 0.816)
Right	47.24 ± 3.70	51.77 ± 4.70	0.768 (0.709, 0.832)	0.752 (0.689, 0.822)
**Calf circumference (cm)**				
Left	26.78 ± 3.87	29.37 ± 4.97	0.841 (0.775, 0.914)	0.840 (0.772, 0.916)
Right	26.80 ± 3.90	29.15 ± 4.43	0.849 (0.781, 0.922)	0.847 (0.778, 0.922)
**Leg length (cm)**				
Left	67.59 ± 4.78	66.73 ± 3.90	1.052 (0.987, 1.12)	1.069 (0.994, 1.150)
Right	67.48 ± 4.78	66.66 ± 3.92	1.049 (0.985, 1.117)	1.067 (0.993, 1.147)
**Leg surface area (cm**^**2**^**)**				
Left	2472.46 ± 319.00	2734.48 ± 377.75	0.998 (0.997, 0.999)	0.998 (0.997, 0.999)
Right	2529.78 ± 304.56	2785.02 ± 402.93	0.998 (0.997, 0.999)	0.998 ( 0.997, 0.999)
**Leg volume (cm**^**3**^**)**				
Left	5000.91 ± 882.68	5932.30 ± 1125.60	0.911 (0.883, 0.940)	0.905 ( 0.874, 0.938)
Right	5024.09 ± 876.43	5960.21 ± 1091.82	0.903 (0.873, 0.934)	0.893 ( 0.860, 0.928)

**Table 3:** Canonical correlation analysis between body measures and chronic diseases.

Body measures variables	ABMS* combination coefficient	SBMS** combination coefficient
Left upper arm circumference	0.00365	− 0.147
Waist width	0.33597	− 0.160
Left thigh circumference	− 0.10713	− 0.194

*ABMS* adverse body measures score; *SBMS* sarcopenia-associated body measures score.

* Canonical correlation coefficient = 0.294476 (p = 0.0026).

ABMS = 0.00365 × Left upper arm circumference + 0.33597 × Waist width − 0.10713 × Left thigh circumference.

**Logistic regression using backward selection method (p < 0.05).

SBMS = 17.3571 - 0.147 × Left upper arm circumference − 0.1595 × Waist width − 0.194 × Left thigh circumference.

**Figure 1:**
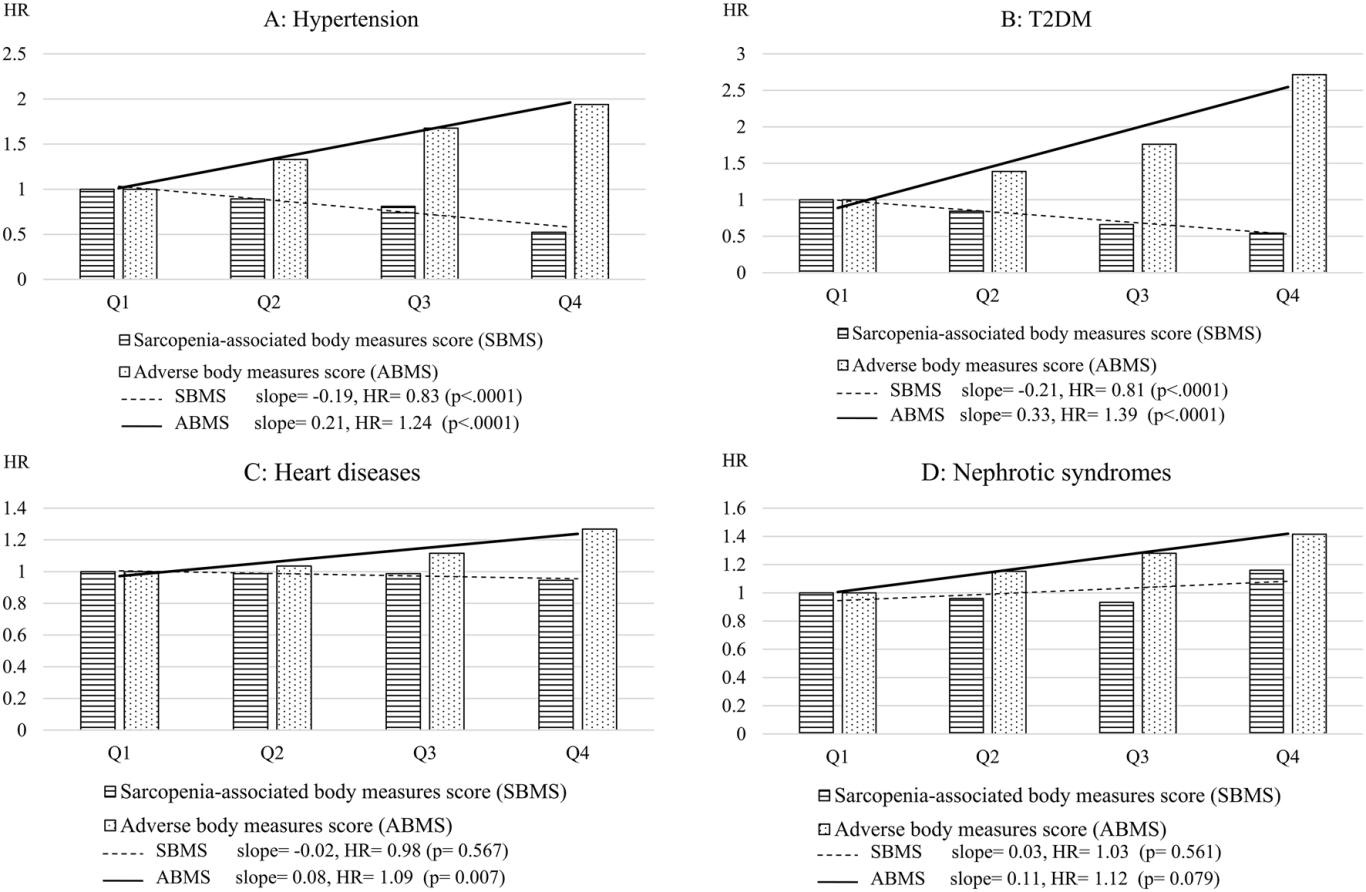
Comparison between sarcopenia-associated adverse body measures score (SBMS) and adverse body measures score (ABMS) in terms of their linear relationship with chronic diseases (the analyses were performed by Cox regression adjusted for age, sex, smoking, alcohol drinking, betel nut chewing, occupation, education, marriage, food style, and personal disease).

We followed up this cohort for 15 years to investigate the association of both the ABMS and SBMS with the selected chronic diseases. Each increment in the ABMS was significantly associated with the hazard ratios of 1.24 (95% CI 1.17–1.30), 1.39 (95% CI 1.29–1.50), 1.09 (95% CI 1.02–1.16), and 1.12 (95% CI 0.99–1.27) for hypertension, T2DM, heart disease, and nephrotic syndrome, respectively. In addition, each increment in the SBMS was associated with the hazard ratios of 0.83 (95% CI 0.79–0.87), 0.81(95% CI 0.76–0.87), 0.98 (95% CI 0.93–1.04), and 1.03 (95% CI 0.93–1.15) for hypertension, T2DM, heart disease, and nephrotic syndrome, respectively. Furthermore, we categorized the ABMS and SBMS into quartiles and found a dose–response relationship between the quartiles and chronic diseases. However, the strength of the association between the SBMS and chronic diseases was less significant (Fig. 1).

## Discussion

Sarcopenia is harmful to human health. Many diseases including obesity^20^, insulin resistance^21^, diabetes^22^, dyslipidemia^23^, and hypertension^24^ are caused by sarcopenia. A study reported that an increased risk of metabolic syndrome in individuals with sarcopenia was linked to weights in upper-normal and slightly overweight ranges^25^. This study further confirmed the association of upper arm circumference, waist width, and thigh circumference with sarcopenia; moreover, the derived adverse body shape, which corresponds to the ABMS rather than muscular loss, results in poor health outcomes. The ABMS, a surrogate for sarcopenic obesity, includes the measurements of larger upper arm circumference and waist width and smaller thigh circumference; thus, the ABMS is superior to sarcopenia alone for predicting chronic diseases.

The results of this prospective cohort analysis indicated that upper arm circumference, waist width, and thigh circumference measurements can be used as alternative biomarkers for predicting sarcopenia-associated chronic diseases, including hypertension, T2DM, heart disease, and nephrotic syndrome, in Taiwan. Patients with sarcopenia generally have smaller measurements of almost all the limbs due to skeletal muscle loss. However, in this study, we observed that smaller measurements of the lower limbs increased the risk of diseases, whereas the smaller measurements of the upper limbs and visceral fat accumulation synergistically increased the risk of diseases. Although studies have indicated that thigh circumference is negatively associated with T2DM, dementia, and cancer^12,16,17^, this study confirmed that it is a long-term predictor of sarcopenia-associated chronic diseases. The present study proposes using the ABMS, which involves the use of upper arm, waist, and thigh measurements for its calculation, as a reliable biomarker for predicting sarcopenia-associated chronic diseases. Thus, the use of the ABMS as an innovative measurement is feasible in preventive medicine and epidemiological surveys in large-population communities.

The lower limbs account for approximately 70% of muscle mass. Studies have indicated that larger hip and thigh circumferences are associated with a low risk of T2DM, dementia, and internal cancers, independent of sociodemographic variables^12,16,17^. The soft tissue in the thigh is mostly composed of muscle mass and subcutaneous fat. The muscle of the lower limbs prevents from diseases in two aspects. First, the muscle absorbs glucose, thus regulating insulin resistance and inflammation to prevent diseases. Second, gluteofemoral body fat may secrete adipokines, including leptin, adiponectin, and inflammatory cytokines, through different actions and feedback systems to prevent diseases. Other studies have also reported an association of a large thigh circumference with a low risk of chronic diseases such as diabetes, hypertension, and heart disease^26,27^. Furthermore, studies have demonstrated an association of a small thigh circumference with hyperglycemia, heart disease, and premature death^28,29^.

WC and WHR are frequently used in current practice to estimate body fat accumulation in the trunk and are biomarkers for many metabolic disorders. A study showed that WC explains obesity-related health risk better than BMI does and that for a given WC value, people have similar health risks^30^. In this study, however, waist width was an indicator of abdominal fat accumulation when upper arm circumference measures were simultaneously considered. A larger upper arm circumference represents body fat accumulation in the upper trunk. Therefore, compared with using waist width alone, using a combination of waist width and upper arm circumference may better indicate upper body fat accumulation in relation to subsequent long-term adverse effects. Our previous study showed that a combination of neck circumference and waist width may be a feasible and comprehensive predictor of long-term diseases such as T2DM^12^. In addition, this study demonstrated that the combination was associated with multiple chronic diseases.

This study proposes that a combination of upper arm circumference, waist width, and thigh circumference is comprehensive, feasible, and superior to sarcopenia-related body measurements alone for predicting chronic diseases including hypertension, T2DM, heart disease, and nephrotic syndromes. A combination of anthropometry measurements and body fat distribution was superior to sarcopenia-related body measurements alone for predicting sarcopenia-associated diseases. Considering that muscle loss in sarcopenia results in poor health outcomes, upper body fat accumulation and lean lower limbs can be vital predictors. Therefore, the measurements proposed in this study to evaluate health outcomes could be more viable than sarcopenia-related body measurements alone in both clinical practice and preventive medicine in the future. The novelty of this study is that it proposes the ABMS as an alternative to the SBMS alone for predicting health outcomes. Clinically, two important aspects were derived from the findings. First, the measurement of lower limb circumference is vital for the identification of individuals with a high risk of chronic diseases. In addition to medications, the management of specific body measurements, including increasing the muscle content in the lower limbs, reducing abdominal fat accumulation, and reducing the fat content in the upper limbs, can be beneficial for preventing chronic diseases.

This study used a two-stage design to obtain body measurements associated with sarcopenia and related them to chronic diseases by linking a long-term follow-up cohort with a national health insurance claims database. The strengths of this study include the use of accurate disease diagnosis data, comprehensive whole-body scanning, and an adequate follow-up duration for cohort analysis. Nevertheless, this study has some limitations. First, this study used a three-dimensional surface scanning technique to obtain body measurements in which muscle mass and fat distribution could not be differentiated. Second, findings were obtained from a community population, which represents the general Taiwan population, and therefore, the findings should be cautiously applied to different ethnic populations. Third, different health care systems may provide diverse intervention plans, leading to different levels of disease risk. In addition, health-seeking behavior, social support, and health literacy may change health outcomes, but nondifferential misclassification was assumed in this study. Finally, interventions such as exercise, nutrition, and medicine can modify body measurements, but obtaining data on these was not feasible in this study.

## Conclusions and implications

Adverse body shape rather than sarcopenia itself causes poor health outcomes. Sarcopenia-associated body measurements, including upper arm circumference, waist width, and thigh circumference, can be used for independently predicting the risk of chronic diseases such as T2DM, hypertension, heart disease, and nephrotic syndrome. Smaller measurements of the lower limbs and larger measurements of the upper limbs and trunk can be useful biomarkers for predicting an individual’s risk of chronic diseases. The body measurements proposed in this study can be used to determine the risk of sarcopenia in clinical practice and preventive medicine.
